# Deconvoluting Substrates, Support, and Temperature Effects on Leaching and Deactivation of Pd Catalysts: An In Situ Study in Flow

**DOI:** 10.1021/acscatal.4c02028

**Published:** 2024-06-13

**Authors:** Oliver J. Newton, Matthew J. Takle, Jeffery Richardson, Klaus Hellgardt, King Kuok Mimi Hii

**Affiliations:** † Department of Chemistry, Imperial College London, Molecular Sciences Research Hub, 82, Wood Lane, London W12 0BZ, U.K.; ‡ Discovery Chemistry Research and Technologies, Eli Lilly and Company, Windlesham, Surrey GU20 6PH, U.K.; § Department of Chemical Engineering, Imperial College London, Exhibition Road, South Kensington, London SW7 2AZ, U.K.

**Keywords:** palladium catalysis, leaching, deactivation, heterogeneous catalysis, Heck reaction

## Abstract

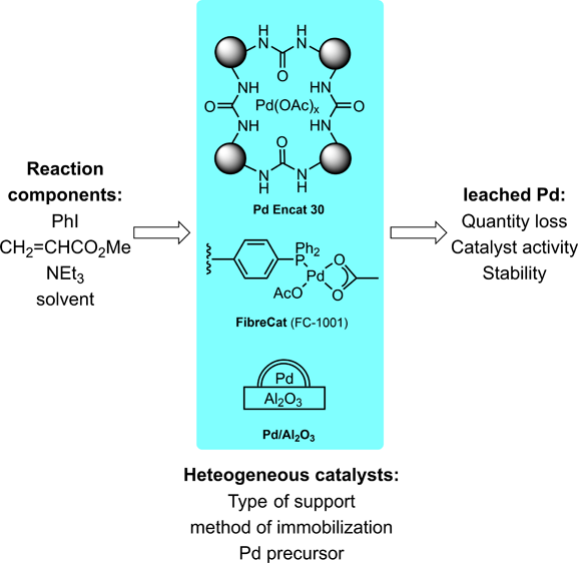

Leaching behavior of three different Pd heterogeneous catalysts (PdEnCat 30, FibreCat FC1001, and Pd/Al_2_O_3_) during the Heck reaction of iodobenzene and methyl acrylate, in the presence of triethylamine, was compared using a tandem flow reactor. While leaching was observed in all three cases, Pd/Al_2_O_3_ appeared to be the most robust, showing little/no leaching at ambient temperature. The leached Pd species also appear to display different catalytic activities. With a slight modification of the reactor, the leaching caused by individual components of the reaction mixture can be assessed separately. For the polymer-supported catalysts, triethylamine caused the largest amount of leaching, even at 30 °C. In contrast, the leaching from Pd/Al_2_O_3_ was observed only in the presence of iodobenzene at 90 °C. Variations in leaching behavior were ascribed to differences in Pd species and immobilization methods.

## Introduction

Palladium-catalyzed C–C bond forming reactions have played a pivotal role in the production of pharmaceutical ingredients^1^ and agrochemicals in the past decades.^2^ The popularity of these methodologies is undoubtedly driven by their efficiency and robustness. However, there are also serious issues with the use of Pd catalysts at scale: in terms of cost and scarcity of platinum group metals (PGMs), engendering concerns with their sustainability.^3^ The toxicity of Pd also causes issues for downstream processes: as heavy metal residues in pharmaceutical products are tightly regulated,^4^ protracted purification procedures are often needed to remove and recover the metal residues, further exacerbating the unsustainability of these processes. In principle, many of these issues can be mitigated by using immobilized catalysts that can be easily separated and recovered from the reaction mixture for reuse.^5^ In practice, however, heterogeneous catalysts generally suffer from low activity, deactivation, and leaching, limiting their use in industrial processes.^6,7^

Indeed, catalyst leaching in cross-coupling reactions, and the role of the leached species in catalytic turnover, have been topics for many debates.^8,9^ Recently, there has been growing evidence to show that the mercury poisoning test is an unreliable method for discriminating between homogeneous and heterogeneous catalysis,^10,11^ casting doubts on earlier studies. Discounting the mercury poisoning test, most leaching studies are conducted by corelating reaction conversions with the amount of Pd present in the reaction aliquot.^12−17^ In comparison, there were fewer studies of catalyst leaching in situ, as these often required customized techniques and reactors. One of the earliest examples is a two-compartment reactor reported by Rothenberg and co-workers,^18,19^ where a nanofiltration membrane was used to physically separate Pd catalyst nanoparticles in a separate compartment from other reaction components: iodobenzene, butyl acrylate, and a heterogeneous base (NaOAc). Observation of Heck coupling product in the solution will therefore imply the leaching of <5 nm Pd species across the membrane. Other studies include the use of X-ray techniques and ICP to directly observe and determine the speciation of Pd species in the homogeneous and heterogeneous phases.^20−23^ While all these studies are effective in detecting leached Pd, their catalytic activity is challenging to determine, as not only dissolved Pd species but also clusters and colloids are known to be catalytically active.^24^ Additionally, none of these studies can totally rule out contributions from heterogeneous catalysis or the involvement of “homeopathic” Pd species that are catalytically active at ppm and even ppb levels.^25−27^

**Figure 1:**
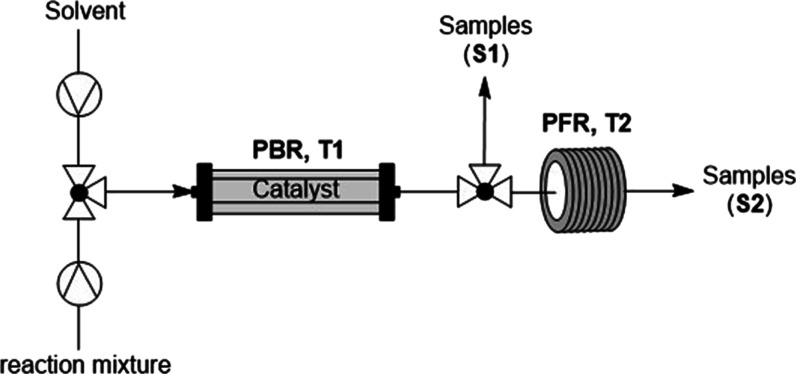
Original configuration of the tandem flow reactor (configuration 1).

**Scheme 1:**
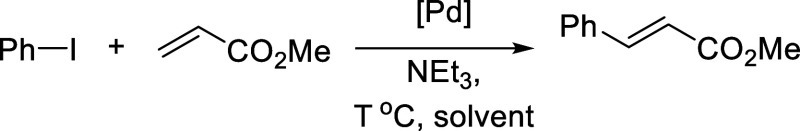
Heck Coupling between Iodobenzene and Methyl Acrylate to Form Methyl Cinnamate

In an earlier paper, we reported the design of a tandem flow reactor system (Figure 1) to study and quantify the leaching behavior of PdEncat 30 during the Heck arylation reaction between iodobenzene and methyl acrylate (Scheme 1).^28^ By measuring the amount of leached Pd (ICP–MS) and comparing reaction aliquots collected after the packed bed reactor (PBR, S1) and plug flow reactor (PFR, S2), it is possible to establish the catalytic activity of the leached species directly. Using multiscale modeling, the contributions of hetero- and homogeneous catalysis to the observed turnover frequency (TOF) can be individually determined and also enabled us to uncover different rates for Pd leaching. Shortly after our publication, a similar split-flow approach was also reported by de Bellefon and co-workers,^29^ which also concluded that Suzuki cross-coupling reactions using Pd/SiO_2_ occur almost exclusively via leached species in the homogeneous phase.

In this paper, we report a systematic examination of the role of each reaction component (solvent, reactants, and base) in leaching of Pd from different supports. The questions we aimed to address are

- Which form of immobilization is most effective in reducing the amount of Pd leaching?
- Which reaction component(s) cause(s) Pd leaching?
- What is the nature of the leached species generated from different types of supports?

## Results and Discussion

**Figure 2:**
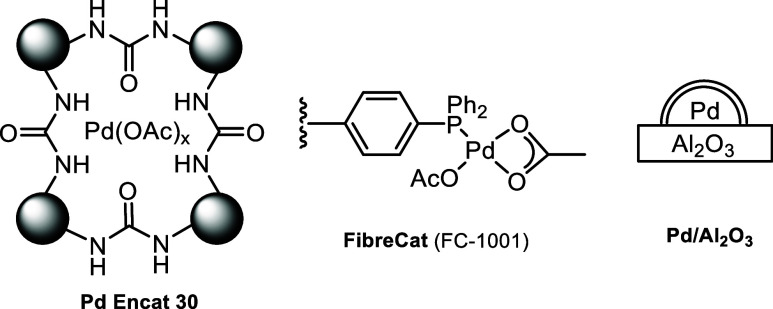
Three heterogeneous Pd catalysts were selected for this study.

Three commercially available heterogeneous Pd catalysts, each representing the most common types of immobilization (Figure 2), were selected for comparison in this study:

(i) PdEncat 30: first reported by Ley et al.,^30,31^ it comprises of Pd(OAc)_2_ encapsulated in a polyurea matrix. It has been shown to act as a reservoir for catalytically active Pd species during the Heck coupling reactions in DMF,^32,33^ and so it was selected to provide a benchmark.^28^
(ii) Pd FibreCat (FC-1001) belongs to a class of supported homogeneous catalyst system, where Pd(OAc)_2_ is immobilized onto a phosphine-functionalized polypropylene polymer.^34^ It was employed in the Heck coupling between 4-cyanoiodobenzene and butyl acrylate in the presence of DIPEA in a flow reactor by Kappe and co-workers,^12^ where leaching of Pd from the packed bed reactor into the reaction mixture was detected at 100 °C in acetonitrile. However, the authors assumed that catalytic activity occurs only on the packed bed.
(iii) Pd/γ-Al_2_O_3_: alumina is one of the most popular metal oxides used to support Pd nanoparticles for cross-coupling reactions,^35−40^ including Heck arylation reactions.^39,40^ In independent studies of Suzuki–Miyaura reactions catalyzed by Pd/Al_2_O_3_, conflicting evidence for^41^ and against^42^ the “heterogeneity” of the reactions have both been presented.

### Solvent Effects

**Figure 3:**
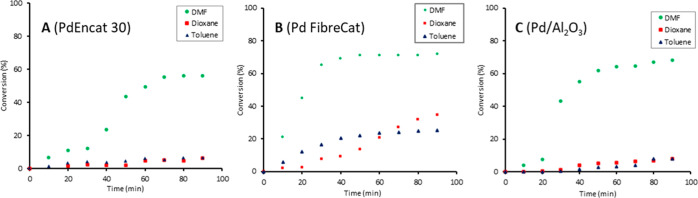
Reaction profiles of Heck coupling obtained using PdEnCat 30, FC-1001, and Pd/Al_2_O_3_ in toluene, dioxane, and DMF in batch reactors. Reaction conditions: Pd catalyst (1 mol %), PhI (3.84 mmol), methyl acrylate (7.40 mmol), NEt_3_ (7.40 mmol), solvent (50 mL), 90 °C. % Conversions to product were determined by HPLC (see S1.1, Supporting Information).

The choice of reaction solvent can have a profound effect on activity of Pd catalysts in cross-coupling reactions.^43,44^ With this in mind, the catalyst activity and stability of PdEnCat 30, FC-1001, and Pd/Al_2_O_3_ were compared in the three most common solvents deployed in Heck reactions: DMF, toluene, and dioxane. To eliminate temperature effects on catalyst activity and stability, the experiments were conducted in parallel batch reactors at 90 °C, below the boiling point of the most volatile solvent (dioxane). Reaction aliquots were extracted every 10 min and subjected to HPLC analysis, affording a reaction profile in each solvent (Figure 3). After 90 min, moderate conversion of between 60 and 70% can be observed in DMF for all three catalysts, accompanied by the development of a strongly colored solution (Figure S3, Supporting Information), signifying the formation of colloidal Pd and Pd black, associated with leaching and catalyst deactivation. The sigmoidal reaction profiles showed an induction period when the active catalyst is generated in situ, which subsequently deactivates. In contrast, very little catalytic turnover (6–7% conversion) was observed with PdEnCat 30 and Pd/Al_2_O_3_ in dioxane and toluene under the same conditions, where the reaction aliquots also remained relatively clear (Figure S3, Supporting Information). By comparison, FC-1001 exhibited a greater extent of conversion to product (up to 25%) in these less polar solvents. These preliminary observations showed that DMF afforded much higher catalytic turnovers, associated with the greatest amount of leaching.

The solvent effect was re-examined in the flow reactors. Using the original configuration (Figure 1), the temperature of the PBR containing PdEncat 30 was set at 90 °C (PBR, T1) and 110 °C for PFR (T2). Reaction mixtures generated in the different solvents were then pumped through the system, and the conversions at S1 and S2 were monitored by HPLC.

**Figure 4:**
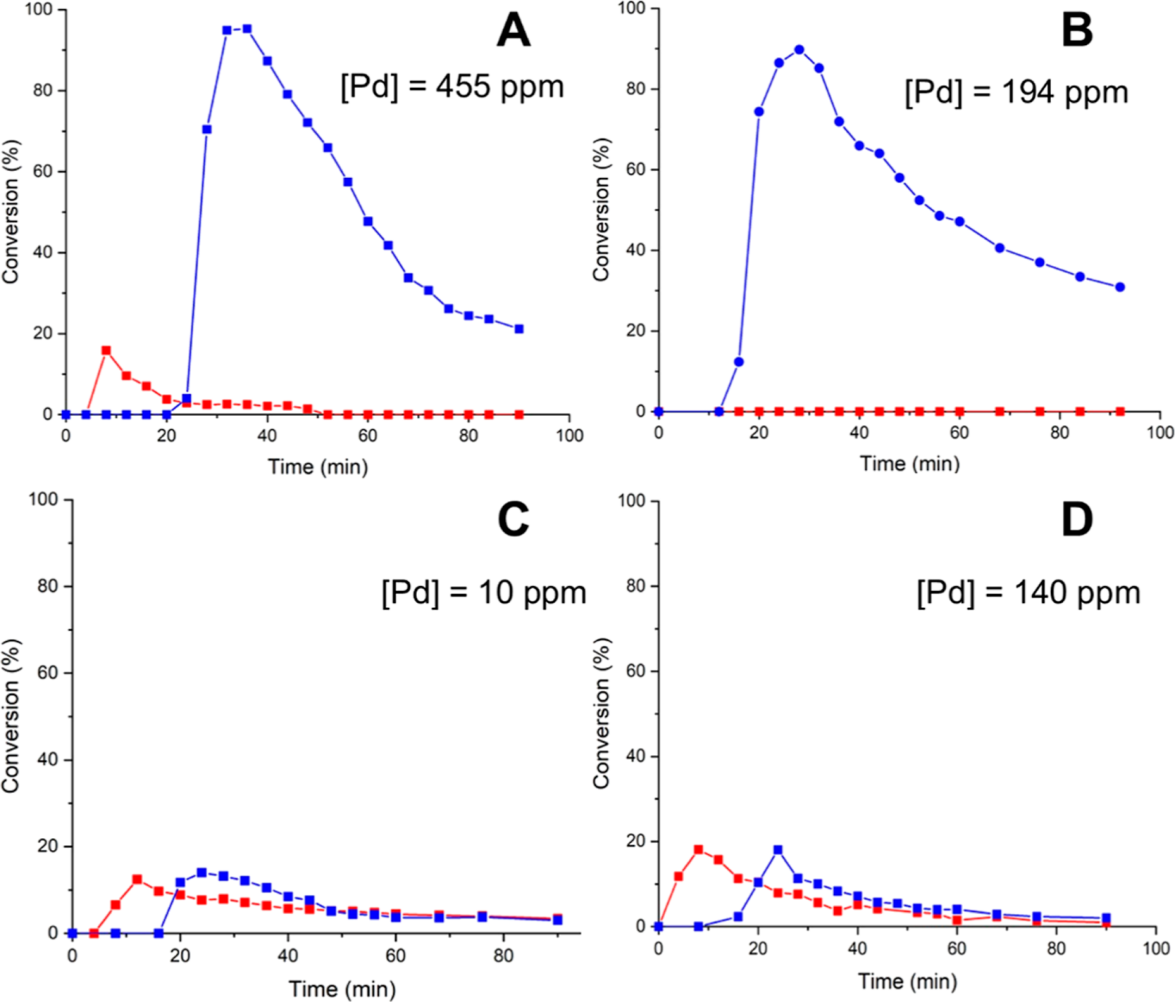
Evaluation of leaching of PdEnCat 30 in different solvents in the tandem flow reactor (configuration 1, Figure 1): (A) DMF (T1 = 90 °C, T2 = 110 °C); (B) DMF (T1 = 30 °C, T2 = 110 °C); (C) dioxane (T1 = 90 °C, T2 = 110 °C); and (D) toluene (T1 = 90 °C, T2 = 110 °C). Red = S1 conversions; blue = S2 conversions.

As reported in our earlier study,^28^ extensive leaching of catalytically active palladium occurs when DMF was employed as the solvent, as indicated by a greater conversion observed at S2 than at S1 (Figure 4A). In this case, the total amount of Pd in solution was found to be 455 ppm, corresponding to 11% of the total amount of Pd present on the pristine catalyst. When T1 was lowered to 30 °C, no product formation could be detected at S1 (Figure 4B). However, the observation of significant catalytic conversion at S2, accompanied by a loss of 194 ppm of Pd to the solution detected by ICP, suggests not only that leaching of active catalyst occurs even under ambient conditions. Comparing the reaction profiles recorded at S2 in Figure 4A,B, a much steeper decrease in catalytic activity can be observed in the former, which we can attribute to a faster deactivation of the active catalyst by agglomeration as a result of a higher concentration of Pd in solution as well as the higher temperature.^26^ In contrast, the leaching of Pd was suppressed in dioxane and toluene. Even at 90 °C, only 10 and 140 ppm of Pd were found in the collected reaction mixtures (corresponding to 0.25 and 3.5% of the supported Pd). More interestingly, identical reaction profiles were observed at S1 and S2 in these cases (Figure 4C,D), showing that the low number of turnovers (<20% conversion) occurred only in the packed-bed reactor and that the leached Pd species are catalytically inactive.

From these initial studies, we can deduce that DMF is involved in generating and maintaining the catalytic activity of the leached Pd species. This is consistent with literature reports, where DMF was known to generate and stabilize Pd nanoclusters that are highly active in cross-coupling reactions.^45−48^

### Nature of Support

**Figure 5:**
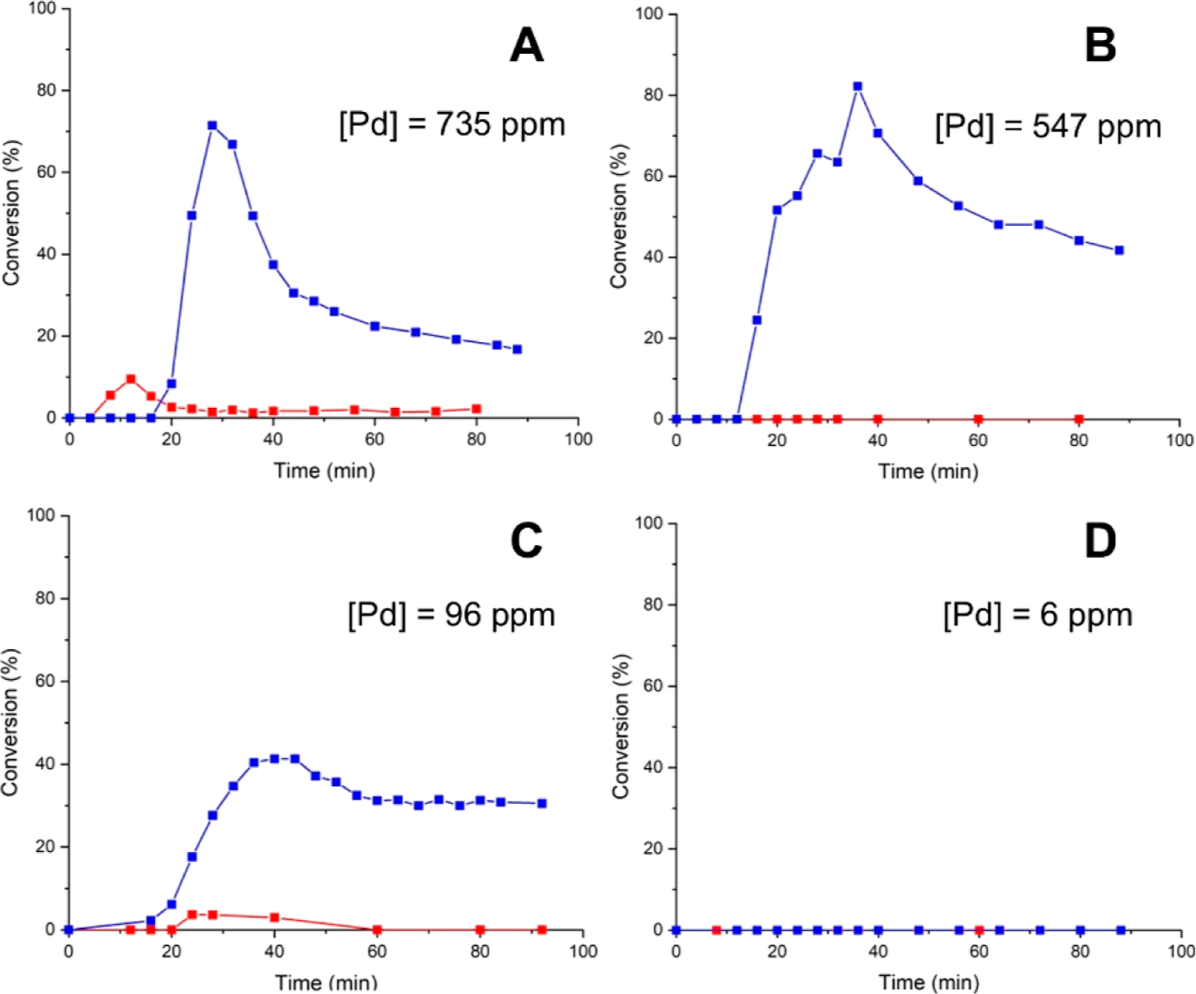
Assessing leaching of FC-1001 and Pd/Al_2_O_3_ at 90 °C (A and C, respectively) and 30 °C (B and D, respectively) in DMF, using a tandem reactor (configuration 1, Figure 1). T2 = 110 °C in all these experiments. Red = S1 conversions; blue = S2 conversions.

Next, the stability of FC-1001 and Pd/Al_2_O_3_ in DMF was similarly studied. As in previous experiments, the same amount of Pd (4 mg/experiment) was deployed in all of the experiments, so the amount of leached Pd between experiments and catalyst supports can be directly compared (see Section S1.2 and Table S1 for precise values). With the temperature of the PBR set at 90 °C, leaching of active catalysts was also observed in both cases (Figure 5A,C). Leaching from the polymer-supported FC-1001 was more pronounced than the microencapsulated PdEncat 30 (735 ppm compared to 455 ppm of [Pd]), leading to a steeper decline in catalytic activity, as indicated by a maximum conversion of <75% at 30 min compared to 100% when PdEncat was employed (Figure 4A). When the temperature of the PBR was lowered to 30 °C, a similar conversion of ca. 80% can be maintained, despite a decrease in the amount of leached palladium to 547 ppm (Figure 5B vs 5A). This is accompanied by a gentler decline in catalyst activity, suggesting leaching of a more stable catalyst.

In contrast, a very different profile was observed for Pd/Al_2_O_3_ (Figure 5C). In this case, the extent of leaching at 90 °C is very reduced (96 ppm). While a maximum conversion of only 40% was reached, the leached species does not appear to deactivate as noticeably compared to the polymer-supported catalysts. A “pseudosteady state” conversion of ca. 30% can be maintained between 60 and 90 min. Subsequently, when the experiment was repeated with T1 = 30 °C, no product formation can be observed at S1 or S2 (Figure 5D), and only 6 ppm of Pd residue was found in the reaction mixture. Hence, we can conclude that the metal oxide-supported Pd nanoparticle is more resistant against leaching than the polymer-supported catalyst in DMF under comparable conditions, although catalytic turnover remained closely associated with leached species.

### Assessment of Individual Reaction Components

**Figure 6:**
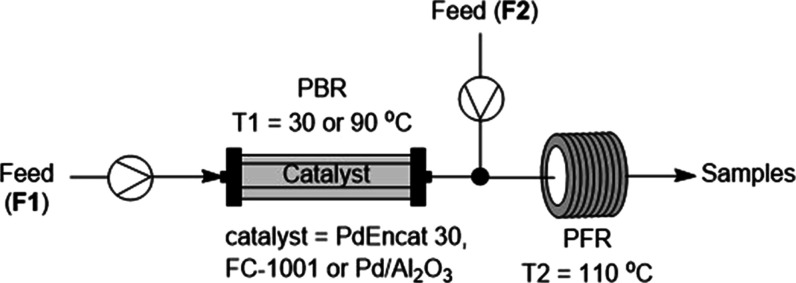
Modified tandem reactor (configuration 2) for monitoring leaching caused by individual reaction components.

In the second part of this work, the tandem reactor system was reconfigured by replacing the sampling point at S1 with an inlet (F2), fed by a syringe pump (Figure 6). Using the modified reactor, it is possible to study the extent of leaching induced by each reaction component and the catalytic activity of the leached species at slightly above ambient (30 °C) and elevated (90 °C) temperatures. In a typical experiment, the catalyst packed bed is exposed to a solution of a specific reaction component via the first pump (F1). Any leached Pd species will be carried by the mobile phase, where it will be mixed with the other reaction components at F2 before passing through the PFR at 110 °C, where the catalytic activity of the leached species can be examined. At the same time, leaching from the PBR at different temperatures was assessed. Each experiment began with the T1 set at a stable “ambient” temperature of 30 °C, where the feeds (F1 and F2) were started and samples collected. After 90 min, the temperature of the PBR was raised to 90 °C, and sample collection continues for a further 90 min. The total amount of Pd residue present in the collected fractions was determined by ICP–MS.

**Figure 7:**
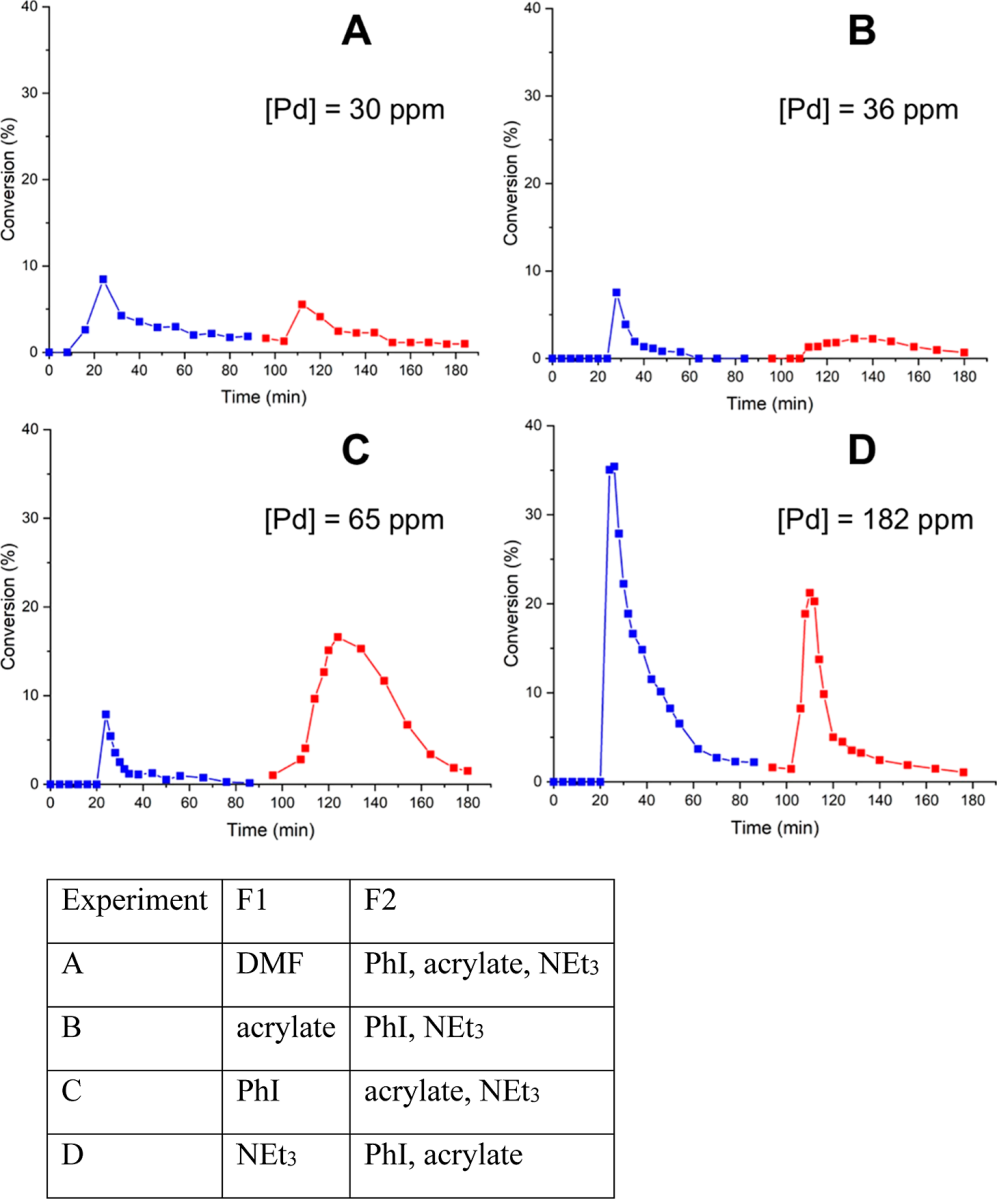
Leaching experiments were conducted with PdEnCat30 using the modified reactor (configuration 2, Figure 6). Each experiment started with PBR (T1) at 30 °C (blue), then increased to 90 °C (red).

A set of 4 experiments were performed with PdEnCat 30 (Figure 7). The first experiment exposes the PBR to “neat” DMF at 30 and 90 °C. Under these conditions, only 30 ppm of Pd was detected in the collected fractions, and very little catalytic activity (<8% conversion) was observed, even when the T1 was raised to 90 °C (Figure 7A). The next experiment with methyl acrylate at F1 yielded similar results to experiment A (Figure 7B), with <8% product formation and a very similar amount of Pd residue found in the collected reaction mixture (36 ppm). Hence, we can deduce that DMF and methyl acrylate cause very little leaching from PdEnCat, nor do they generate active catalysts. This suggests that DMF is likely to serve as a stabilizer to retain the catalyst activity of the leached species, although it does not cause any leaching itself.

Next, the catalyst was exposed to iodobenzene, which has been widely implicated for causing leaching.^15,49−51^ At 30 °C, a very similar level of catalytic activity to experiments A and B was observed (Figure 7C). However, at 90 °C, significant product formation was observed, signifying leaching of active catalyst, which was supported by a higher amount of Pd residue in the collected fractions (65 ppm). This observation supports the earlier hypothesis that aryl iodide causes the leaching of active catalyst by oxidative addition of ArI to Pd(0), which only takes place at elevated temperatures. However, the amount of leached palladium, compared to that observed in previous experiments, was somewhat lower than what might be expected.

**Scheme 2:**
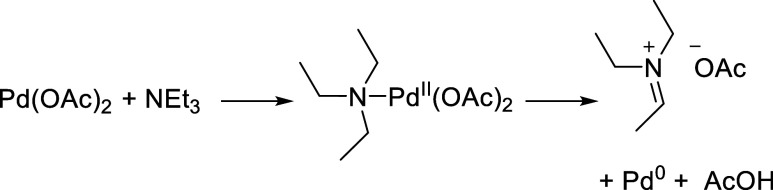
Reaction of Pd(OAc)_2_ with Triethylamine to Form Pd(0) via a Dehydrogenative Mechanism

The final experiment yielded a surprising result of this study (Figure 7D): even at a mild temperature of 30 °C, exposure of PdEnCat 30 caused the highest amount of catalytically active Pd species to be generated in the liquid phase. This was rather unexpected; while tertiary alkylamines are known to interact with Pd(II) compounds to form Pd(0) species via an α-hydride elimination pathway (Scheme 2),^52^ this was known to occur only at high temperatures. For example, Pd(OAc)_2_ reacts with tributylamine at 100 °C to generate Pd(0).^53^ Comparing the amount of Pd leached (182 ppm) with that previously observed for catalyst leaching by the reaction mixture at the same temperature (Figure 4B, 194 ppm), we can conclude that NEt_3_ is the biggest culprit for causing leaching.

**Figure 8:**
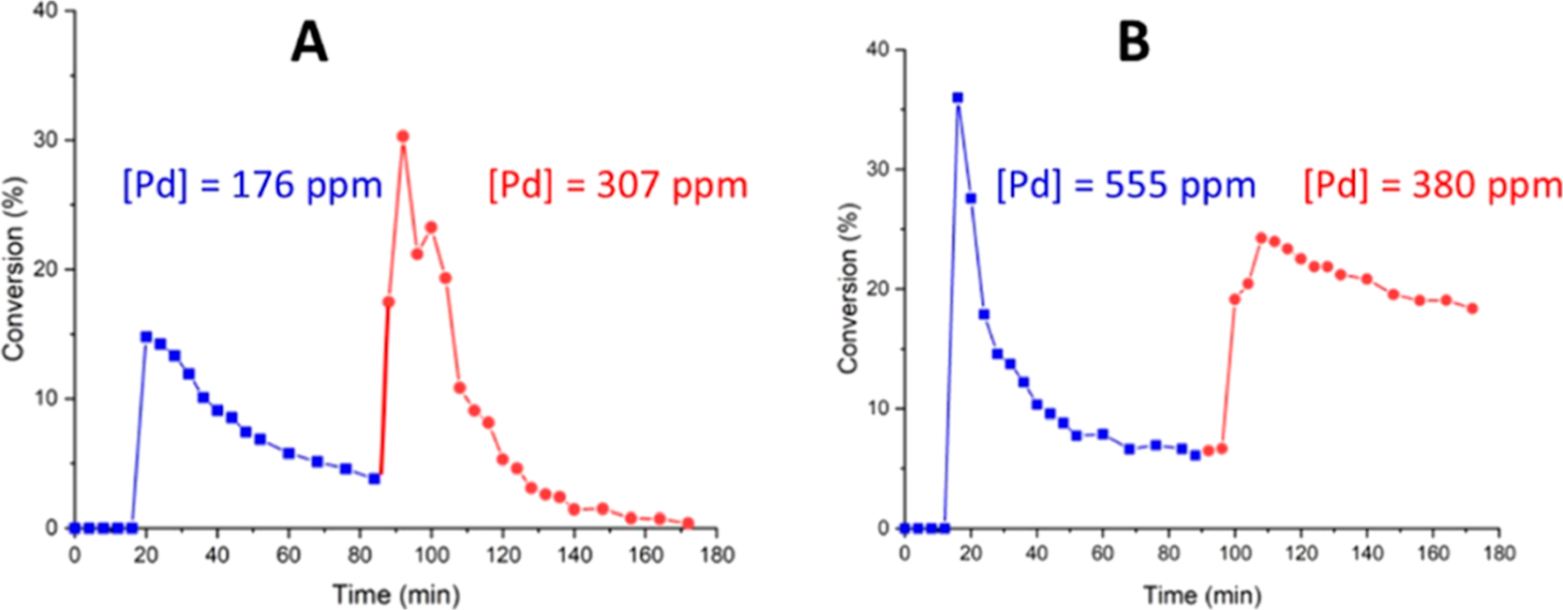
Pd leaching from FC-1001 by PhI (A) and NEt_3_ (B). Each experiment started with PBR (T1) at 30 °C (blue), then increased to 90 °C (red).

The studies were replicated with the phosphine-anchored FC1001. In this case, the Pd residue in the fractions collected at 30 and 90 °C was determined separately. Compared to PdEnCat 30, both iodobenzene and triethylamine caused significant leaching of active catalysts at 30 °C (Figure 8), with the latter causing the greatest amount of leaching (555 compared to 176 ppm). Comparing the higher-temperature profiles of Figure 8A with 8B, a similar amount of Pd was collected, but a steeper reduction in catalytic activity was observed in the leached species generated by PhI compared to that generated by triethylamine, which has a gentler decline. We believe this indicates that different Pd species are leached when FC-1001 is exposed to iodobenzene or NEt_3_, and that the greater stability of the latter is due to the coordination of the trialkylamine to Pd(0), effectively acting as a stabilizer against agglomeration.

**Figure 9:**
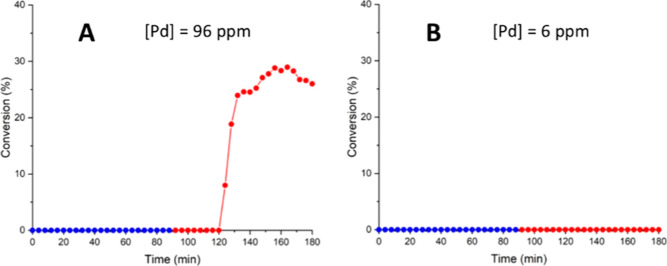
Stability of Pd/Al_2_O_3_ in iodobenzene (A) and triethylamine (B). Each experiment started with PBR (T1) at 30 °C (blue), then increased to 90 °C (red).

Last but not least, a drastically different leaching behavior was observed for Pd/Al_2_O_3_ compared to that of the polymer-supported catalysts. In contrast to the first two catalysts, Pd is immobilized as nanoparticulates, consisting of a thin layer of PdO on the surface. In this case, leaching of an active catalyst was observed only in the presence of iodobenzene at 90 °C (Figure 9A). In this case, ICP analysis of the collected reaction aliquots revealed a Pd content of only 96 ppm with no visible sign of deactivation for 60 min. In contrast, the metal oxide-supported Pd nanoparticles were found to be completely stable in the presence of triethylamine at both 30 and 90 °C (Figure 9B). No catalytic activity was observed when the nanoparticles are exposed to the Lewis base; only 6 ppm of [Pd] was detected in the reaction mixture, providing an interesting counterpoint to our earlier studies with polymer-supported catalysts (Figures 7D and 8B). It is also interesting to compare this to one of our earlier studies,^23^ where a mixture of an inorganic base (K_2_CO_3_) in ethanol was found to cause significant leaching from Pd/Al_2_O_3_ under mild conditions, which we attributed to an attack of the base on the alumina support. This highlights that the choice of a base can have an important effect on catalyst leaching.

By far, this appears to be the only system where the interaction with the aryl iodide is the only reaction component that causes leaching. The mechanisms by which ArX can cause leaching from surfaces of Pd nanoparticles have been extensively studied computationally by the research groups of Heinz,^54^ Ananikov,^55^ and Köhler.^13^ Based on these reports, we speculate that nature of these leached species is likely to be Pd clusters, which have greater catalyst activity and stability than the molecular species leaching from the polymer-supported PdEnCat and FC-1001.

## Conclusions

The leaching behavior of three different heterogeneous Pd catalysts during the Heck arylation reaction was systematically compared using tandem flow reactors. Overall, polymer-supported Pd(OAc)_2_ (EnCat, FibreCat) was found to be less stable against leaching than the metal oxide-supported Pd/Al_2_O_3_, as quantified by ICP analyses of Pd residues in the mobile phase.

Although significant Pd leaching and catalyst turnover was only observed in DMF with all three catalysts (Figure 3), the solvent itself is not the major culprit (Figure 7A), suggesting that cooperative effects between the reaction components are at play. By exposing the catalyst bed to individual reaction components, their effects on the extent, as well as the catalytic activity, of the leached species can be revealed.

For polymer-supported Pd(II) catalysts, triethylamine was found to cause a greater amount of leaching than iodobenzene, even at ambient temperature, in the absence of catalytic turnover. We attribute this to the Lewis basicity of the amine to coordinate and reduce the Pd(II) to Pd(0) via a dehydrogenative mechanism (Scheme 2). In contrast, leaching of Pd/Al_2_O_3_ was observed only at an elevated temperature (Figure 9A); the metallic nanoparticles were completely stable in the presence of triethylamine, even at elevated temperature (Figure 9B).

It is interesting to note that the formation of Pd black from all three catalysts was observed in experiments conducted in batch reactors (Figures 3 and S3). This suggests that leaching may also depend on how the catalysis was performed: by conducting the leaching studies in a single-pass continuous flow, the residence time in the packed bed reactor is relatively short, and the leached Pd species is not able to redeposit back onto the support. Hence, any catalytic activity due to “boomerang” catalysis^56^ or leaching caused by accumulated products and byproducts, particularly halide salts,^57^ is not likely to be significant under these experimental conditions.

Collectively, these experiments provided valuable insights into the catalyst leaching process, particularly the role of the various reaction components and the type of leached species that they produce from various supports. These findings will guide future catalyst design and optimization of the reaction conditions to minimize Pd loss.

## Supporting Information Available

Experimental methods and details, including analytical methods, description of the flow reactor, and experimental procedures; calculations of ICP result and amount of leached Pd; schematic of configuration 1 and 2 of the tandem flow reactor; and breakthrough curves (PDF)

## Abbreviations

PBR: packed bed reactor
PFR: plug flow reactor
DMF: *N*,*N*-dimethylformamide
ICP–MS: inductively coupled plasma mass spectrometry
