# Supplementary material for: Deconvoluting Substrates, Support, and Temperature Effects on Leaching and Deactivation of Pd Catalysts: An In Situ Study in Flow
Source: ACS Catal. 2024 Jun 13;14(13):9678–86. doi: 10.1021/acscatal.4c02028 (PMC11232010; doi:10.1021/acscatal.4c02028)
Supplement: Supplementary file 1 — cs4c02028_si_001.pdf [file cs4c02028_si_001.pdf]

Supporting information

Deconvoluting substrates, support and temperature effects on leaching and deactivation of Pd catalysts: an in-situ study in flow

Oliver J. Newton,[a,Ψ] Matthew J. Takle,[a] Jeffery S. Richardson,[b,§] Klaus Hellgardt[c] and King Kuok (Mimi) Hii[a,*].

[a] Department of Chemistry, Imperial College London, Molecular Sciences Research Hub, Imperial College London, 82, Wood Lane, London W12 0BZ, U.K.

[b] Former affiliation: Discovery Chemistry Research and Technologies, Eli Lilly and Company, Windlesham, Surrey, GU20 6PH, U.K.

[c] Department of Chemical Engineering, Imperial College London, Exhibition Road, South Kensington, London SW7 2AZ, U.K.

*mimi.hii@imperial.ac.uk

Ψ Current Affiliation: GlaxoSmithKline, Gunnels Wood Road, Stevenage, SG1 2NFX, U.K.

§Current Affiliation: Sai Life Sciences Limited, Alderley Park, Macclesfield, SK10 4TG, U.K.

S1 Materials and characterization methods

Unless stated otherwise, all chemicals and solvents were procured from commercial vendors and were used as received without further purification.

S1.1 HPLC analysis (Fig. 3). HPLC separations were performed on an Agilent 1100 instrument consisting of an G1322A degasser, G1311A quaternary pump, G1313A ALS, G1315B diode array detector (Agilent, USA) and fitted with a Nucleosil C18 column (100-5C18, 12.5 cm × 4.0 mm, 5 µm). Absorbances at 210, 254 and 280 nm were used for quantification. Unless stated otherwise, a gradient HPLC method was used with the mobile phase consisting of solvent A (acetonitrile acidified with 0.1% TFA) and solvent B (Water acidified with 0.1% TFA) with a flow rate of 1.0 mL/min. The initial mobile phase composition was maintained at 50% solvent A for 0.5 min, changed linearly to 95% (0.5–6.5 min), followed by a return to the initial conditions (6.5–7.5 min). The injection volume was 5 µl. Samples were prepared using a 1:50 dilution with acetonitrile containing the internal standard cumene (0.001 mM). Chemstation software (version number: B.04.02 SP1) was used to process and analyze all resulting chromatograms.

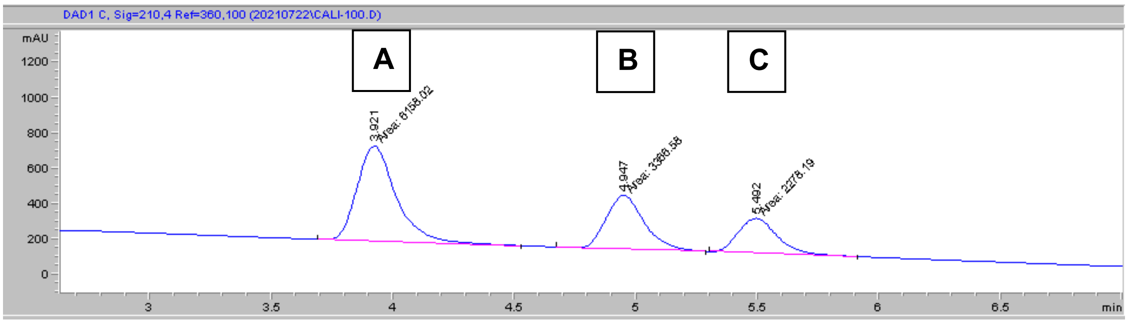

Figure S1: Chromatogram of a reaction aliquot showing the elution of the peaks in the following order – A) Methyl cinnamate, B) Iodobenzene, C) Cumene (internal standard).

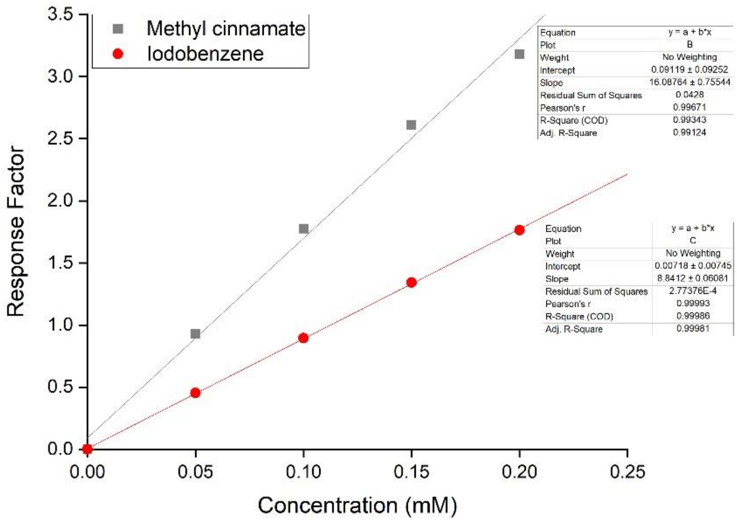

Figure S2: Calibration curves constructed from the response factors of iodobenzene and methyl cinnamate.

S1.2 Inductively coupled plasma (ICP). The same amount of Pd (4.0 mg/experiment) was used in all the experiments. Total metal content in the collected liquid samples was determined by ICP-MS using an Agilent 7900 instrument calibrated with solutions containing 0, 50, 100, 250 and 500 ppb of Pd, prepared from a Palladium ICP/DCP standard solution (10,000 µg/mL Pd in 6% wt.% HCl), which was diluted to the required standards with a 2% nitric acid solution. All samples were digested in aqua regia and diluted with a 2% nitric acid solution to maintain a homogeneous sample.

An example of the calculation of the amount of leached Pd is given below.

PdEnCat30 used for flow reactions = 95 mg

Quantity of elemental Pd in 95 mg of PdEnCat30 (0.4mmol/g) = 0.038 mmol

Mass of elemental Pd in 95 mg of PdEnCat30 = 0.038 mmol * 106.42 g/mol = 4.0 mg

Subsequently, 80 mg of 5% Pd/Al_2O_3 and 100 mg of 3.97% Pd assay FibreCat 1001 were deployed accordingly.

Dilution procedure for samples:

Maximum amount of possible Pd leaching = 4 mg (4000 μg) for 100% leaching.

The samples were digested in aqua regia before being diluted 1000 fold to give a max amount of 4 ug/mL (4 ppm or 4000 ppb)

Result e.g.

100 ppb (ug/L) determined by ICP –> 100 ppb (ug/L) in 1 L dilution = 100 ug Pd leached (0.10 mg)

(0.10 mg leached/ 4 mg initial)*100 = 2.5 % Pd leached.

Table S1. Calculations of ICP result and amount of leached Pd.

ICP results ppb (ug/L)	ppm (mg/L): accounting for 1000 fold dilution	Leached Pd (mg)	% leached from initial Pd (4mg loading)
100	100	0.1	2.5
455	455	0.455	11.375

10	10	0.01	0.25
194	194	0.194	4.85
140	140	0.14	3.5
547	547	0.547	13.675
735	735	0.735	18.375
96	96	0.096	2.4
6	6	0.006	0.15
30	30	0.03	0.75
36	36	0.036	0.9
65	65	0.065	1.625
182	182	0.182	4.55
176	176	0.176	4.4
307	307	0.307	7.675
555	555	0.555	13.875
380	380	0.38	9.5

S2. Methods

S2.1 Comparison of solvents in batch reactors (Fig. 3). The comparison of catalyst leaching in batch reactors was performed in parallel: Each of the 100 mL round bottomed flasks was charged with the either PdEnCat30 (95 mg) or 5% Pd/Al_2O_3 (80 mg) (corresponding to 1 mol %) followed by 50 mL of DMF, toluene or dioxane. Iodobenzene (430 µL, 3.84 mmol), methyl acrylate (670 µL, 7.4 mmol) and triethylamine (1.02 mL, 7.4 mmol) were added to generate the reaction mixtures. Three reaction mixtures, each generated in a different solvent, were heated to 90 °C in parallel using a DrySyn® parallel synthesis kit with magnetic stirring. Reaction aliquots (20 µL) were extracted every 10 minutes over a 90 minute period, which were diluted with 1 mL of a 1 mM solution of cumene (internal standard) in MeOH, before subjected to HPLC analysis (section S1.1).

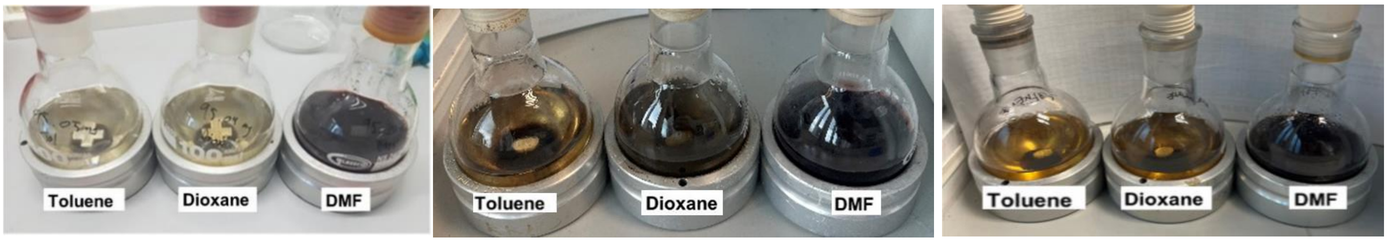

Figure S3: Appearance of reaction mixtures after 90 minutes. Left: reactions with PdEnCat30. Middle: reactions with Pd FibreCat. Right: Reactions with Pd/Al_2O_3.

S2.2 Preparation of catalyst pack beds. Catalysts (1 mol%, 4 mg Pd content) were dispersed within quartz sand (1 g, <250 µm, Fisher), prior to being packed into a PFA tube (100 x 4 x 6 mm length x inner Ø x outer Ø, Adtech) sealed at either end with glass wool (Figure S1). The packed tube was inserted into the aluminium block, and two silicon O-rings (Polymax) were fitted to either end of the cartridge to create a seal between the PFA tube and the Swagelok fittings.

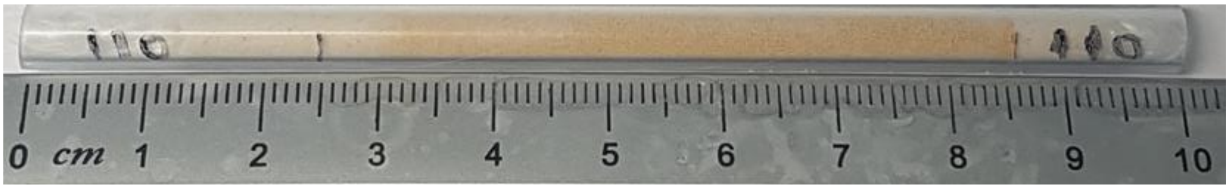

Figure S4: Picture of the packed catalyst cartridge.

S2.3 Tandem Flow Reactor: Configuration 1

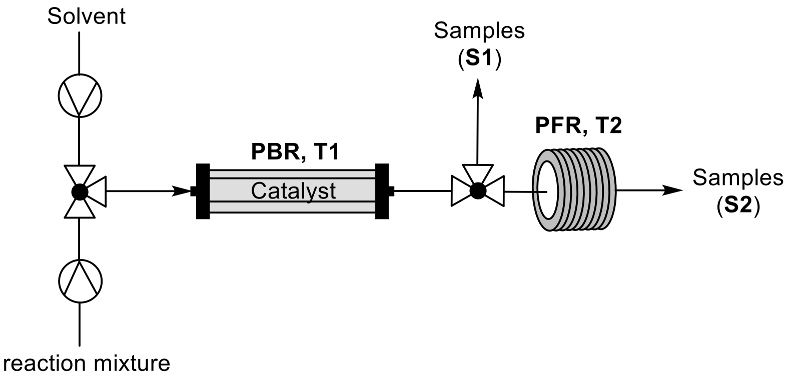

Figure S5: Schematic of configuration 1 of the tandem flow reactor.

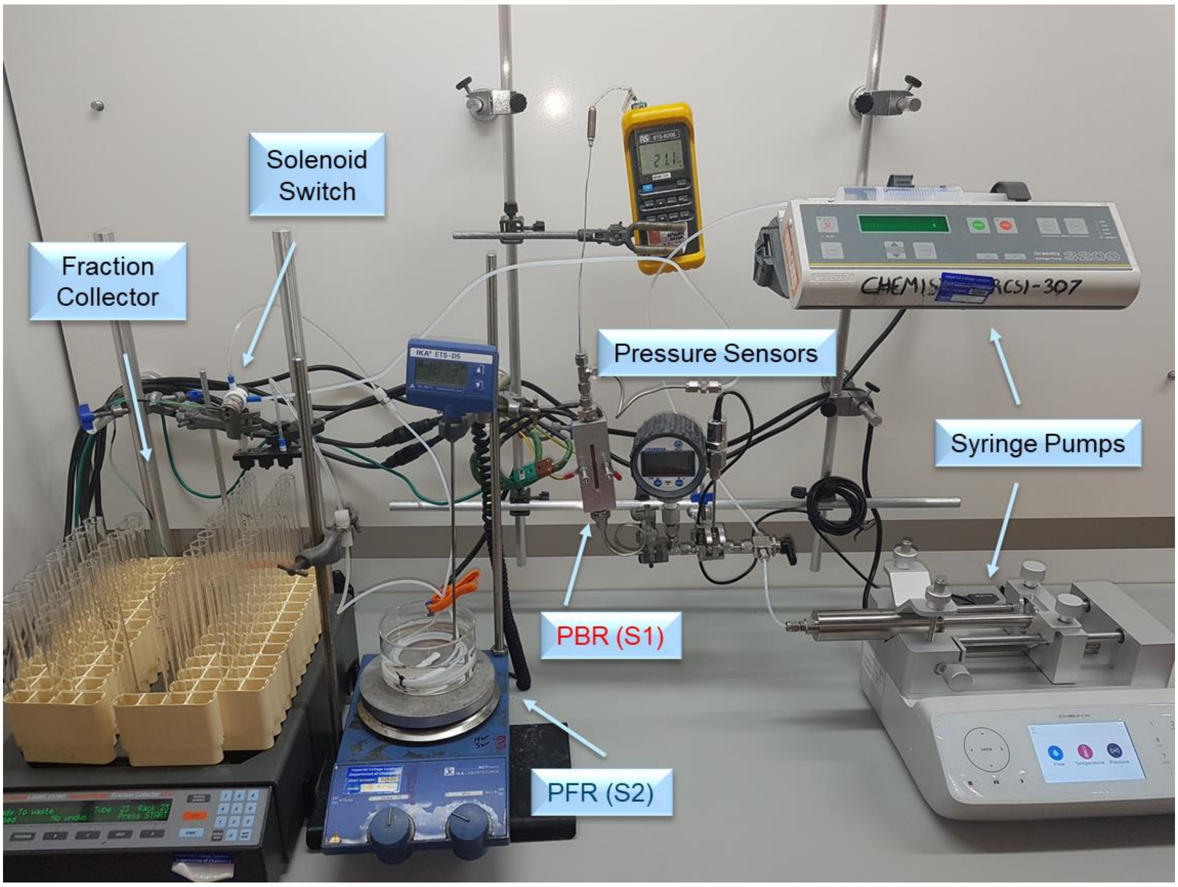

Figure S6: An image of the tandem flow reactor set up in configuration 1.

In configuration 1, the solvent or reagents are delivered to the reactor via a high pressure syringe pump (Chemyx fusion 6000 fitted with a 50 mL stainless steel syringe) through a 3-way ball valve. An additional syringe pump (Graseby 3100, fitted with a 50 mL plastic syringe), containing the reaction solvent, is used to purge the reactor initially. The pressure of the system is monitored using a pressure gauge (Omega®) positioned between the 3-way ball valve and the entrance of the PBR. The PBR is an aluminium block (102 x 35 x 20 mm) with drilled cylindrical bores which fit the PFA catalyst cartridge (100 x 4 x 6 mm length x inner Ø x outer Ø, Adtech) and heater cartridges (1/4" x 3", Under Control Instruments Ltd). The heater cartridges are powered and controlled with a PID controller (Sesto D1S-VR-200). A K-type thermometer is fitted to the side of the PBR which provides temperature feedback to the PID controller. Both ends of the PBR are fitted with Swagelok fittings connecting to 1/8" metal tubing. At the top of the PBR a K-type thermometer is fitted through a T-piece to measure the temperature of the fluid immediately exiting the PBR. The outlet of the T-piece is connected to a solenoid valve which splits the flow between a Spectra/Chrom® CF-2 Fraction Collector (S1) and a coil of 1/8" PFA tubing (PFR, 1 m, internal Ø = 1/16"). The PFR is submerged in a silicone oil bath, heated with a hot plate with independent heating controls. The exit of the PFR is connected to the fraction collector (S2) which is programmed to collected samples at set

intervals. Each sample is analysed for reactant and product concentration and then all samples are combined and reduced using a BioChromato Smart Evaporator and subjected to ICP analysis for total Pd content.

S2.4 Standard Experimental Procedure for Configuration 1

A PFA catalyst cartridge (100 x 4 x 6 mm length x inner Ø x outer Ø, Adtech) was packed (section S2.2) with the desired heterogeneous Pd catalyst (1 mol %, 4 mg Pd content) and inserted into the PBR. The heating rods attached to the PID controller were then inserted into the PBR along with the feedback k -type thermocouple. A 50 mL syringe was filled with the desired solvent and loaded into pump 1 (Graseby 3100 syringe pump). A second 50 mL syringe was filled with a solution containing iodobenzene (420 µL, 3.76 mmol), methyl acrylate (670 µL, 7.4 mmol) and triethylamine/DIPEA (7.4 mmol) made up to 50 mL with the desired solvent and loaded into pump 2 (Chemyx fusion 6000 high pressure syringe pump). Both syringes were then connected to the reactor via the 3-way ball valve. Prior to the start of each reaction the temperature was set to 25 °C on both the PID controller and on the oil bath. The system was flushed through with the solvent from syringe pump 1 at a flow rate of 0.5 mL/min. The solenoid valve was switched off to direct the flow to S2 only. Once the system is filled, syringe pump 1 was stopped and the 3-way ball valve closed. If required, the temperatures of the PBR and PFR were set to their desired value. Once the desired temperatures had been reached, the 3-way ball valve was opened to the direction of syringe pump 2 which was then set to a flow rate of 0.5 mL/min. The solenoid valve was then switched on (50 Hz), to split the flow between S1 and S2. The eluent was collected using a Spectrum Chromatography® CF-2 fraction collector which was programmed to the desired time interval. Fractions from S1 and S2 were analysed using HPLC to quantify the formation of methyl cinnamate and any remaining reactants. Fractions from S1 and S2 were combined, reduced and then subjected to ICP analysis for Pd content.

S2.5 Tandem Flow Reactor: Configuration 2

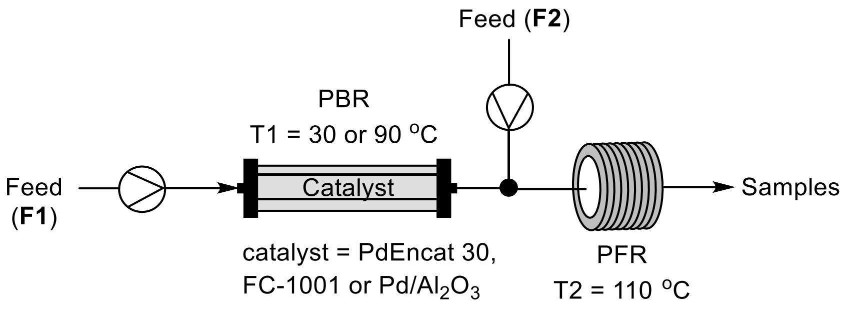

Figure S7: Schematic of configuration 2 of the tandem flow reactor.

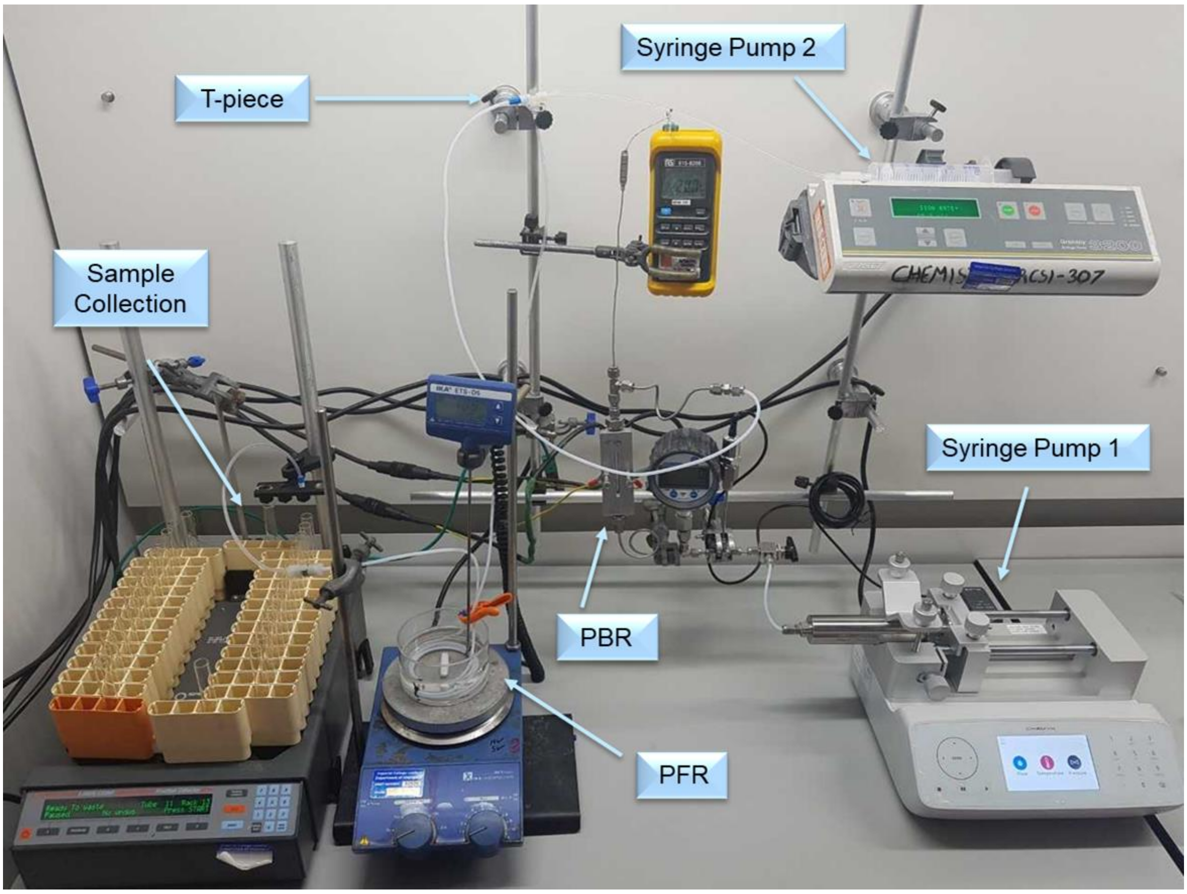

Figure S8: An image of the tandem flow reactor set up in configuration 2.

The individual solvent or a mix of solvent and reagents are delivered to the reactor via a high pressure syringe pump (Chemyx fusion 6000 fitted with a 50 mL stainless steel syringe) through a 3-way ball valve. The pressure of the system is monitored using a pressure gauge (Omega®) and a pressure sensor positioned between the 3-way ball valve and the entrance of the PBR. The PBR is an aluminium block (102 x 35 x 20 mm) with drilled cylindrical bores which fit the PFA catalyst cartridge (100 x 4 x 6 mm length x inner Ø x outer Ø, Adtech) and heater cartridges (1/4” x 3”, Under Control Instruments Ltd). The heater cartridges are powered and controlled with a PID controller (Sesto D1S-VR-200). A K-type thermometer is fitted to the side of the PBR which provides temperature feedback to the PID controller. Both ends of the PBR are fitted with Swagelok fittings connecting to 1/8” metal tubing. At the top of the PBR a K-type thermometer is fitted through a T-piece to measure the temperature of the fluid immediately exiting the PBR. The outlet of the T-piece is connected to another T-piece which mixes the flow from the exit of the PBR with the remaining reactants for the Heck reaction delivered through an additional syringe pump (Graseby 3100 fitted with a 50 mL luer lock Norm-Ject syringe). The exit stream of the T-piece is connected to a coil of 1/8” PFA tubing (PFR, 1 m, internal Ø = 1/16”). The PFR is submerged in a silicon oil bath sitting on a hot plate with independent heating controls. The exit of the PFR is connected to a Spectra/Chrom® CF-2 Fraction Collector (S2) which is programmed to collected at set intervals.

S2.6 Standard Experimental Procedure for Configuration 2

A PFA catalyst cartridge (100 x 4 x 6 mm length x inner Ø x outer Ø, Adtech) was packed (Section 2.2) with the desired heterogeneous Pd catalyst (1 mol %, 4 mg Pd content) and inserted into the PBR. The heating rods attached to the PID controller were then inserted into the PBR along with the feedback k -type thermocouple. Prior to the start of each reaction the temperature was set to 25 °C on both the PID controller and on the oil bath. The system was then purged at a flow rate of 0.5 mL/min with solvent from pump 1 (Chemyx fusion 6000 high pressure syringe pump). Once the system is filled, syringe pump 1 was stopped and the 3-way ball valve closed. Syringe pump 1 was then loaded with a 50 mL stainless steel syringe filled with a solution containing one out of the three components for the Heck reaction being either iodobenzene (420 µL, 3.76 mmol), methyl acrylate (670 µL, 7.4 mmol) or triethylamine/DIPEA (7.4 mmol)

made up to 50 mL with the desired solvent. A 50 mL plastic syringe was filled with a solution containing the remaining two components of the Heck reaction and made up to 50 mL in the desired solvent and loaded into pump 2 (Graseby 3100 syringe pump). If required, the temperatures of the PBR and PFR were set to their desired value. Once the desired temperatures had been reached, the 3-way ball valve was opened and then set to a flow rate of 0.25 mL/min. Syringe pump 2 was simultaneously set to a flow rate of 0.25 mL/min to deliver a total flow rate of 0.5 mL/min through the PFR. The eluent was collected using a Spectrum Chromatography® CF-2 fraction collector which was programmed to the desired time interval. Fractions were analysed using HPLC to quantify the formation of methyl cinnamate. Fractions were combined, reduced and then subjected to ICP analysis for Pd content.

S2.7 Flow Reactor Profile - Breakthrough Curve

A series of breakthrough curves were carried out using the tandem flow reactors at different flow rates. Breakthrough curves are important to establish the residence time and the reactor volume.

First, DMF was passed through the flow reactor at a flow rate of 2 mL/min to fill the reactor volume. Then a solution of Iodobenzene in DMF was passed through the flow reactor at a flow rate of 0.5 mL/min with fractions being collected at 1 min intervals. The concentration of iodobenzene was quantified using HPLC analysis. Breakthrough curves were performed for both S1 and S2 outlets (Figures S8 & S9), showing plug flow behaviour.

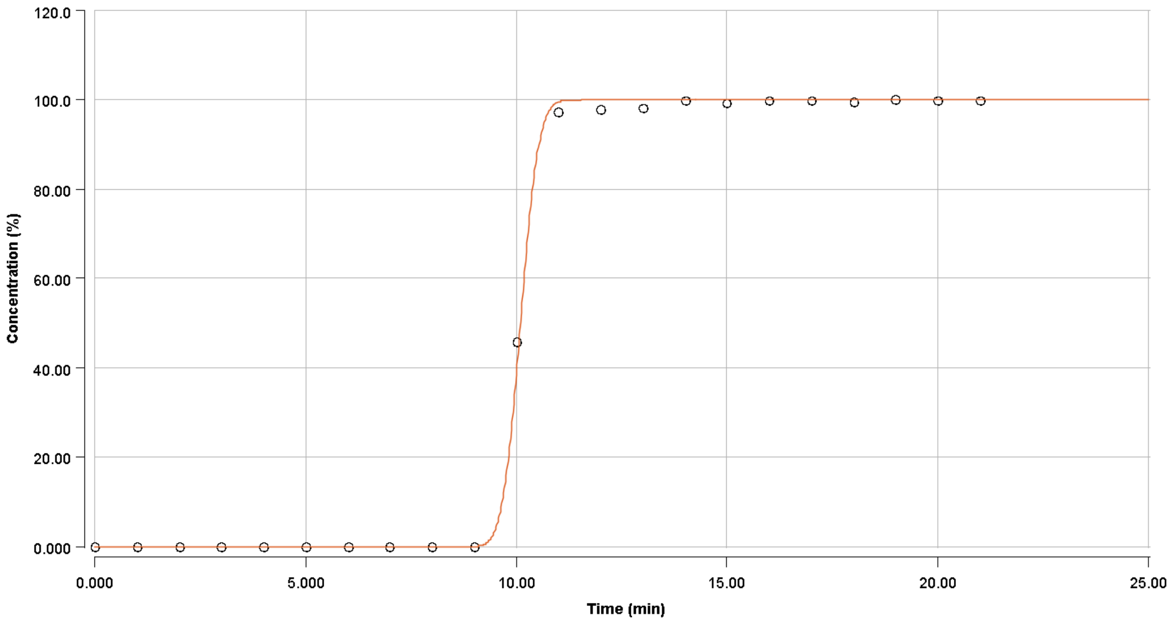

Figure S9: Breakthrough curve for the flow reactor eluting from S1 at 0.5 mL/min.

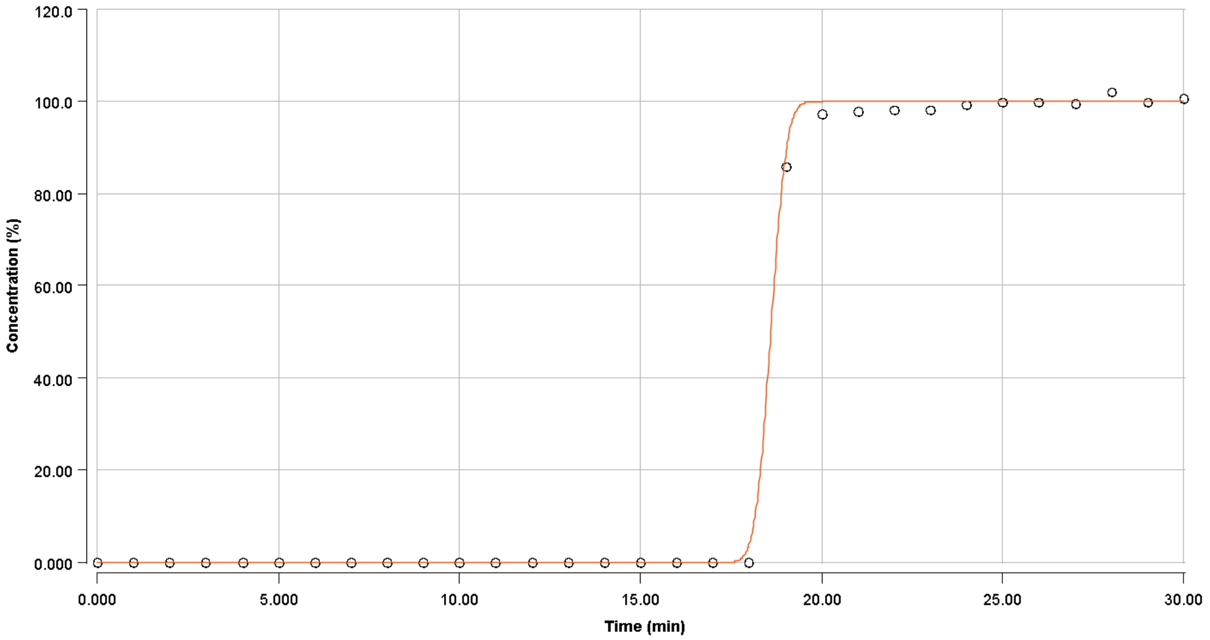

Figure S10: Breakthrough curve for the flow reactor eluting from S2 at 0.5 mL/min.
